# Religious hallucinations in Lebanese patients with schizophrenia and their association with religious coping

**DOI:** 10.1186/s13104-023-06296-0

**Published:** 2023-03-02

**Authors:** Rabih Fares, Jean-Marc Rabil, Chadia Haddad, Sami Helwe, Joe Khalil, Carina Kasrine Al Halabi, Tiffany Abi Antoun, Georges Haddad, Souheil Hallit

**Affiliations:** 1 School of Medicine and Medical Sciences, Holy Spirit University of Kaslik, P.O. Box 446, Jounieh, Lebanon; 2 Research Department, Psychiatric Hospital of the Cross, Jal Eddib, Lebanon; 3 School of Health Sciences, Modern University of Business and Science, Beirut, Lebanon; 4 INSPECT-LB (Institut National de Santé Publique, d’Épidémiologie Clinique et de Toxicologie- Liban), Beirut, Lebanon; 5 Applied Science Research Center, Applied Science Private University, Amman, Jordan

**Keywords:** Religious hallucinations, Psychotic symptoms, Schizophrenia, Lebanon

## Abstract

**Purpose:**

to evaluate the relationship between religious hallucinations and religious coping among Lebanese patients with schizophrenia.

**Methods:**

We have studied the prevalence of religious hallucinations (RH) among 148 hospitalized Lebanese patients suffering from schizophrenia or schizoaffective disorder in November 2021 exhibiting religious delusions (RD), and their relationship with religious coping using the brief Religious Coping Scale (RCOPE). The PANSS scale was used to evaluate psychotic symptoms.

**Results:**

After adjustment over all variables, more psychotic symptoms (higher total PANSS scores) (aOR = 1.02) and more religious negative coping (aOR = 1.11) were significantly associated with higher odds of having religious hallucinations, whereas watching religious programs (aOR = 0.34) was significantly associated with lower odds of having religious hallucinations.

**Conclusion:**

This paper highlights the important role of religiosity that has to play in the formation of religious hallucinations in schizophrenia. Significant association was found between negative religious coping and the emergence of religious hallucinations.

## Introduction

Many people with mental health disorders seek help in times of crisis in religion and spirituality. This concept was originally founded in human societies that have been shaped by diverse religions that provided hope, emotional support, and welfare [1]. However, it is still nowadays challenging for the mental health field to be able to distinguish between normal religious experiences and pathological ones, since spirituality belongs to the individual’s subjective dimension of experience, and religious delusions (RD)/ religious hallucinations (RH) need to be socially framed in order to be clinically evaluated which is often challenging for mental health professionals [2].

Lebanon is one country with the most spiritually and culturally diverse population in the Middle East region, consisting of one third of Christians and two-third of Muslims (including mainly Druz, Shia and Sunni Muslims). As a result, the Lebanese national identity was defragmented and replaced on account of different religious background that tend to determine Lebanese people’s identity [3]. This can highlight the importance of religion in shaping the life of Lebanese people as a source of emotional support in times of crisis, as shown in a recent study [4]. In fact, 67.8% of Lebanese people have higher public stigma toward mental health [5] and tend to seek psychological well-being from religion instead. As a result, one may hypothesize that religious themed psychological abnormalities may be prevalent among Lebanese patients with mental health disorders.

Religious themes were frequently observed in delusional disorders [6] and, a recent metanalysis showed that RD/RH were frequently encountered in common major mental illnesses [7]. The DSM 5-TR define religious delusions as the preoccupation of the individual with irrational beliefs that are not within the expected belief of one’s cultural and educational background. These preoccupations may imply religious practices that were shown to be more prevalent among patients with schizophrenia [8]. Since schizophrenia is a psychotic disorder characterized by alterations in thinking, emotional responsiveness and behavior, psychological processes such as perceptions (hallucinations) and reality testing (delusions) may also be disturbed.

Hearing voices are the main component of sensory hallucinations in schizophrenia. These voices could be related to one or many persons, peers, or God and are frequently identified as controlling and commanding the patient’s actions. These auditory hallucinations can sometimes be directly connected to the delusional theme or work as a trigger of developing delusional thoughts, and patients with schizophrenia clearly distinguish these voices from their own inner speech as originating from the external world. One hypothesis illustrates that corollary discharge dysfunctions, which are motor signals that influence sensory processing, could possibly play a role in the development of auditory hallucinations in schizophrenia, since patient’s own inner speech could not be distinguished from external world stimuli [9]. As a result, patients may sometimes refer religious inner speech as being heard from on outside supernatural agents such as God or the devil. On the other hand, paranoid delusions are typical in schizophrenia, and may particularly include persecution, mystic and grandiose themes. In fact, religion and delusions in schizophrenia are severely affected by sociocultural variables [10] and may sometimes connect beliefs that are not established on solid intersubjective reality [11], which may strengthen the loss of contact with reality. For instance, Muslim patients may pretend to be Mohammed, and Christian patients may believe they are Jesus. These alterations may profoundly alter patient’s everyday life functioning, including religious beliefs and practices [8], which are not found when dealing with patients with schizoid or schizotypal personality disorder who can be more aware of the difference between their distorted ideas and reality.

Although the treatment of schizophrenia is based mainly on antipsychotic medication with psychological and social support, the evaluation of the religious background of patients is still underestimated by clinicians [12]. Religious background does not imply only religiousness but also integrates the spirituality in one’s life with all the transcendence and life’s meaning that comes with it. In fact, many patients suffering from schizophrenia focus on their spirituality in time of crisis instead of sharing their difficulties with psychiatrists [7], considering that their religious belief may help them in rebuilding a positive identity despite the disease, to give hope and meaning to their lives, to manage their symptoms, and to find social support and motivation [13]. For instance, one Lebanese study found a positive correlation between spirituality and lower suicidality rates among 159 patients suffering from schizophrenia [14]. Another systematic literature search through 30 years revealed that religiosity could play a role in protecting or emphasizing the expression of neuropathological symptoms in schizophrenia [15].

On the other hand, the risk of experiencing RD/RH in schizophrenia was significantly increased in patients with strong religious activity [16]. In addition, one observational study conducted in India showed that patients with RD and/or RH in schizophrenia tend to have a longer duration of untreated psychosis and present with a more severe illness [17]. Another Mexican study showed that patients with schizophrenia who exhibited RD/RH had more psychiatric hospitalizations and poorer psychosocial functioning when compared to those with no history of RD/RH [18]. At a first glance, one may consider that RD/RH prevalence may be higher in the Arab World rather than Western Countries, due to the social importance that still holds religion when dealing with emotional distress and inappropriate behavior [19]. However, the prevalence of RD/RH vary slightly in prevalence among countries, ranging similarly among Western civilizations, principally influenced by the Judeo-Christian religion, and the Arab world, principally influenced by the Islamic religion. In fact, one third of patients with schizophrenia reported RD through the progress of their diseases in the Netherlands [6] and Germany [16], approximately equivalent to that found in Egypt [20] and Turkey [21]. Therefore, the prevalence of developing RD/RH was not influenced by the underlying religion.

In contrast, Mishra et al. [17] showed that religious coping was significantly associated with the development of RD/RH, with more prevalence in those who read or watch religious themed activities and adopt personal prayers alone or before meals. Another recent study among 14 females in Qatar showed that half of the participants are highly religious and reported auditory hallucinations such as a call for prayer (Azan) when having psychotic-like experience [22]. Considering the important role of religiosity in shaping mental health, the coalescence of religion and culture [14] and the lack of studies assessing such relationships in Lebanon, we found it important to evaluate a potential relationship between RH and religious coping among Lebanese patients with schizophrenia or schizoaffective disorder.

## Methods

### Participants and study design

**Fig. 1:**
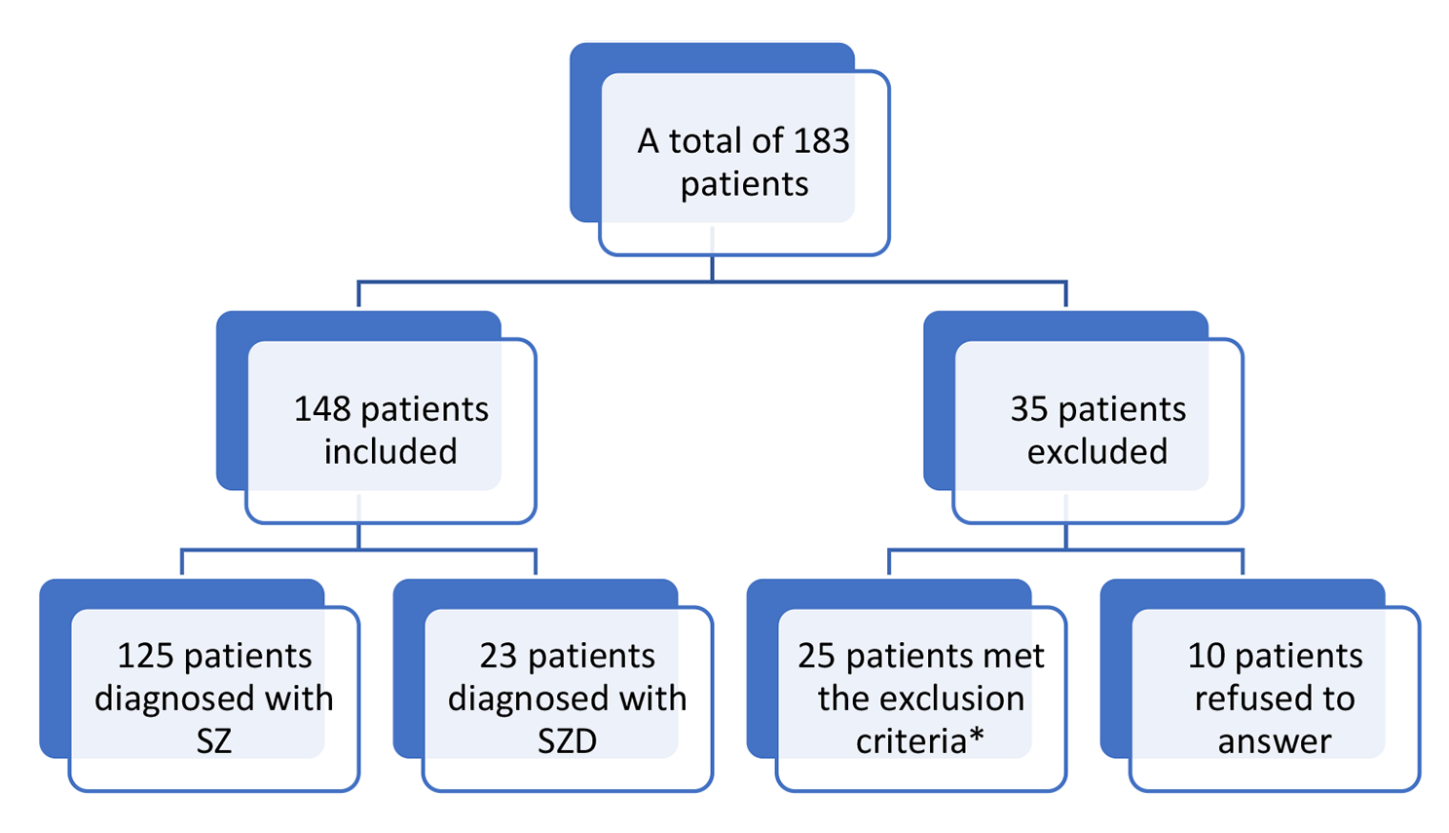
Flowchart showing the process of our sample selection. 183 patients at PHC had the diagnosis of Schizophrenia (SZ) or schizoaffective disorder (SZD) according to DSM-V standards. *25 patients met the exclusion criteria and had a concomitant Major Neurocognitive Disorder (n = 3), age > 85 (n = 4) or a significant cognitive impairment (n = 18)

A cross sectional study was conducted at the Psychiatric hospital of the Cross, Lebanon in November 2021. A total number of 183 patients aged between 18 and 45 years with the diagnosis of schizophrenia and schizoaffective disorder as per the Diagnostic and Statistical Manual of Mental Disorders, 5th edition DSM-5, hospitalized for more than 1 year and clinically stable (after checking their medical records), were included in this study. The medical files of all the hospitalized patients suffering from schizophrenia or schizoaffective disorder were checked. Only patients with schizophrenia or schizoaffective disorder were included if they exhibited religious delusions, primarily evaluated with the Scale for the Assessment of Positive Symptoms (SAPS) (detailed in the following section). Written informed consent was obtained before the start of the interview. All participants were advised that they can decline participation at any time and withdraw from the study. Exclusion criteria were patients with schizophrenia or schizoaffective disorder who have a history of substance use disorder (except nicotine or caffeine) and suffering from any neurological or comorbid psychiatric disorder, including mental retardation (Fig. 1).

### Procedure

Questions covered different aspects; the first part included socio-demographic characteristics such as gender, age, educational level, religion, duration of illness and duration of hospitalization. The second part included religious characteristics information such as whether they engage in personal prayers, the reading of religious books and prayers before and after meals. To assess religious delusions, we used the question in the Scale for the Assessment of Positive Symptoms (SAPS) [23]. “The patient is preoccupied with false beliefs of a religious nature”. A score of 2 or more indicated the presence of religious delusions. Since the literature lacks a precise scale that assess religious hallucinations, we ought to evaluate the presence or absence of religious hallucinations on a five-point Likert scale (0 = Never, 1 = A little, 2 = Moderate, 3 = Severe and 4 = Very severe). Those options were recoded afterwards into two categories, 0 and 1 indicating the absence of religious hallucinations, and 2, 3 and 4 indicating the presence of religious hallucinations. Trained medical students were responsible for the data collection, via a personal interview with each patient.

The third part included the following scales:

Positive and Negative Syndrome Scale (PANSS), validated in Arabic [24] was used to assess the severity of psychotic symptoms [25]. It consists of 30 items divided into 3 categories: 7 items assessing positive symptoms, 7 items assessing negative symptoms, and 16 items assessing general psychopathology symptoms. Rating was based on 7 varying levels ranging from “absent” to “extreme”. The total score is calculated by summing the results for each question; the potential ranges are 7–49 for the positive and negative scales and 16–112 for the general psychopathology scale. A higher score indicates a higher severity of symptoms.

The religious coping assessment of the participants was evaluated using the brief Religious Coping Scale (RCOPE) in its Arabic version, a 14-item scale of religious coping with major life stressors [26]. Seven items assess positive religious coping, and seven items assess the negative religious coping. The positive religious coping subscale of the Brief RCOPE taps into a sense of connectedness with a transcendent force, a secure relationship with a caring God, and a belief that life has a greater benevolent meaning [26]. The negative religious coping subscale of the Brief is characterized by signs of spiritual tension, conflict and struggle with God and others, as manifested by negative reappraisals of God’s powers, demonic reappraisals, spiritual questioning and doubting, and interpersonal religious discontent [26]. The scale was scored by summing the positive items and then summing the negative items to create two subscale scores.

### Statistical analysis

SPSS software v25 was used for the analysis. Logistic regression was used to check for an association between presence/absence of religious hallucinations and all other variables. We first calculated the unadjusted odds ratio between one independent variable and the presence/absence of religious hallucinations; then, we calculated the adjusted odds ratio between the independent and the dependent variable after adjusting over all other independent variables in the database. P < 0.05 was deemed statistically significant.

## Results

The prevalence of religious hallucinations was 21.6%. Most patients were females (68.24%), Christians (68.24%), and suffering from schizophrenia (84.45%). Among religious practices, 84.45% of participants had personal prayers, 62.16% were watching religious programs, 52.7% reading religious books, and 74.32% had prayers before meals. No statistically significant association was found between exhibiting RH and age, gender, education level, religion (Christian vs. Muslim), diagnosis (schizophrenia vs. schizoaffective disorder), duration of treatment or duration of hospitalization. After adjustment over all variables, more psychotic symptoms (higher total PANSS scores) (aOR = 1.02) and more religious negative coping (aOR = 1.11) were significantly associated with higher odds of having religious hallucinations, whereas watching religious programs (aOR = 0.34) was significantly associated with lower odds of having religious hallucinations (Table 1).

**Table 1:** Bivariate analysis of factors associated with the presence/absence of religious hallucinations

Variable	Absence of religious hallucinations (N = 116)	Presence of religious hallucinations (N = 32)	*OR [95% CI]*	*aOR [95% CI]*
**Sex**			0.86 [0.38–1.97]	0.88 [0.32–2.44]
Males	36 (76.6%)	11 (23.4%)		
Females	80 (79.2%)	21 (20.8%)		
**Education level**				
Primary	33 (76.7%)	10 (23.3%)	1	1
Complementary	28 (71.8%)	11 (28.2%)	1.30 [0.48–3.50]	1.46 [0.48–4.48]
Secondary	23 (82.1%)	5 (17.9%)	0.72 [0.22–2.38]	0.76 [0.19–3.08]
University	32 (84.2%)	6 (15.8%)	0.62 [0.20–1.90]	0.69 [0.20–2.37]
**Religion**			1.16 [0.51–2.67]	0.87 [0.34–2.22]
Christian	80 (79.2%)	21 (20.8%)		
Muslim	36 (76.6%)	11 (23.4%)		
**Diagnosis**			0.73 [0.23–2.32]	0.85 [0.25–2.94]
Schizophrenia	97 (77.6%)	28 (22.4%)		
Schizo-affective	19 (82.6%)	4 (17.4%)		
**Personal prayers**			0.99 [0.34–2.92]	2.25 [0.54–9.40]
No	18 (78.3%)	5 (21.7%)		
Yes	98 (78.4%)	27 (21.6%)		
**Watching religious programs**			0.53 [0.24–1.16]	**0.34 [0.12–0.94]**
No	40 (71.4%)	16 (28.6%)		
Yes	76 (82.6%)	16 (17.4%)		
**Reading religious books**			1.15 [0.53–2.51]	1.44 [0.57–3.64]
No	62 (79.5%)	16 (20.5%)		
Yes	54 (77.1%)	16 (22.9%)		
**Prayers before and after meals**			0.70 [0.30–1.65]	0.64 [0.22–1.90]
No	28 (73.7%)	10 (26.3%)		
Yes	88 (80.0%)	22 (20.0%)		
**Age (years)**	55.47 ± 12.98	50.75 ± 13.88	0.97 [0.95-1.00]	0.97 [0.94–1.01]
**Duration of treatment (years)**	16.48 ± 11.91	15.63 ± 10.20	0.99 [0.96–1.03]	0.97 [0.91–1.04]
**Duration of hospitalization (years)**	11.04 ± 11.64	11.35 ± 10.56	1.00 [0.97–1.04]	1.05 [0.98–1.12]
**PANSS total score**	61.87 ± 23.29	71.69 ± 26.28	**1.02 [1.001–1.03]**	**1.02 [1.001–1.04]**
**Religious positive coping**	20.57 ± 6.87	19.50 ± 6.84	0.98 [0.92–1.04]	0.96 [0.88–1.04]
**Religious negative coping**	11.22 ± 3.82	13.13 ± 5.33	**1.10 [1.01–1.20]**	**1.11 [1.004–1.23]**

OR refers to adjusted ratio without adjustment over other variables (bivariate analysis). aOR refers to the adjusted odds ratio over all other variables in the table (multivariable analysis). Numbers in bold reflect significant associations (p < 0.05)

## Discussion

Since religion may act as a symptom-formation factor in schizophrenia when dealing with RD/RH [27], our results may suggest that assessing the spirituality and religiosity in patients with schizophrenia or schizoaffective disorder is an essential tool for understanding the patient as a whole, while taking care of his/her needs and struggles [28], especially in a country with many cultural and religious diversity such as Lebanon. To our knowledge, this is the first study in Lebanon that highlighted the impact of religiosity on RH among patients with schizophrenia or schizoaffective disorder and pointed up the importance of considering patients’ religious background when assessing RH in schizophrenia or schizoaffective disorder.

Our results showed that 32 patients (21.6%) displayed RH regardless of their religion. This number may not be very high since most Lebanese people hold religious beliefs that accept hallucinatory experiences as mystical experiences [29], which might explain the lower number of religious themed thoughts and experiences deemed as “hallucinatory” in our sample. However, regarding religiosity and severity of the psychosis, we have found a significant association between having negative religious coping and suffering from RH. In fact, believing in a punitive God resulted in an increase in positive psychiatric symptoms among patients with schizophrenia, whereas a significant decrease in the same symptoms was found when believing in a more benevolent God [30]. Pietkiewicz et al. [31] explained such negative religious coping as a refuge for patients with schizophrenia to interpret their unusual experiences.

Religious practices were prevalent among the participants, since the Lebanese society is highly religious [3]. This high religious activity was also found among patients with schizophrenia showing RD/RH in the Arab World, mainly in Egypt [20] and Turkey [21]. Previous research examining the religiosity in individuals with psychosis showed association between exhibiting hallucinations and religiosity [32]. The authors suggested an explanation where religious practices are adopted in response to RH as a method of coping with their psychosis; a similar situation could be surmised to be happening in our sample, whereby patients are adopting Religious Negative coping because of their psychosis. As for the association between watching religious programs and the decrease of RH, our study showed that watching religious programs can be seen as a protective factor from developing RH, thus, emphasizing more research in the future for a better understanding of this process.

### Implications for mental health care

Patients with religious convictions in schizophrenia may exhibit RD/RH, and mental health professionals (psychiatrists, psychologists, nurses) may argue that their repressed beliefs may be the direct cause of their mental health deterioration. Such an assumption among mental health professionals may induce personal internalized stigma experienced by patients with schizophrenia, negatively affecting their adherence and attitude toward the treatment. Misunderstanding the cultural background of patients with schizophrenia may lead to poor communications and ethical dilemmas, thus, inducing practice problems among mental health professionals. Our study emphasizes on the importance of knowing the spiritual and religious coping practices among patients with schizophrenia presenting RD/RH since negative religious coping may be predictive of developing religious themed hallucinations. Therefore, emphasizing on the participation in spiritual activities by psychiatric health care professionals that encourage positive religious coping mechanisms may decrease positive symptoms in schizophrenia, mainly religious hallucinations.

### Limitations

One limitation could be that all patients were recruited from one hospital, which may predispose us to a selection bias. In addition, we did not go deeper in each subgroup of each religion. Residual confounding bias is also possible since other factors related to religious hallucinations were not considered in this paper.

## Conclusion

Despite these limitations, our study showed a significant association between a negative religious coping and the appearance of religious hallucinations. Clearly, further research needs to be undertaken with a larger sample size to clarify the association between religiosity and the development of religious hallucinations in a religiously diverse country such as Lebanon.

## Data Availability

The authors do not have the right to share any data information as per their institutions’ policies.
